# Acute MDPV Binge Paradigm on Mice Emotional Behavior and Glial Signature

**DOI:** 10.3390/ph14030271

**Published:** 2021-03-16

**Authors:** Mafalda Campeão, Luciana Fernandes, Inês R. Pita, Cristina Lemos, Syed F. Ali, Félix Carvalho, Paulo Rodrigues-Santos, Carlos A. Fontes-Ribeiro, Edna Soares, Sofia D. Viana, Frederico C. Pereira

**Affiliations:** 1 Institute of Pharmacology and Experimental Therapeutics/IBILI, Faculty of Medicine, University of Coimbra, 3000-548 Coimbra, Portugal; mmscampeao@gmail.com (M.C.); lucy4fernaz_@hotmail.com (L.F.); inespita1989@gmail.com (I.R.P.); fontes.ribeiro@gmail.com (C.A.F.-R.); edna.fps@gmail.com (E.S.); sofia_viana@estescoimbra.pt (S.D.V.); 2 Department of Psychiatry, Psychotherapy and Psychosomatics, Division of Psychiatry I, Medical University Innsbruck, 6020 Innsbruck, Austria; a.cristinalemos@gmail.com; 3 Department of Pharmacology, University of Arkansas for Medical Sciences (Adjunct Professor), Little Rock, AR 72205, USA; Syed.Ali@fda.hhs.gov; 4 REQUIMTE, Laboratory of Toxicology, Faculty of Pharmacy, UCIBIO, University of Porto, 4050-313 Porto, Portugal; felixdc@ff.up.pt; 5 Center for Neuroscience and Cell Biology (CNC), Laboratory of Immunology and Oncology, University of Coimbra, 3004-504 Coimbra, Portugal; paulo.santos@fmed.uc.pt; 6 Faculty of Medicine, Institute of Immunology, University of Coimbra, 3004-504 Coimbra, Portugal; 7 Center of Investigation in Environment, Genetics and Oncobiology (CIMAGO), Faculty of Medicine, University of Coimbra, 3004-504 Coimbra, Portugal; 8 Faculty of Medicine, Coimbra Institute for Clinical and Biomedical Research (iCBR), University of Coimbra, 3000-548 Coimbra, Portugal; 9 Center for Innovative Biomedicine and Biotechnology (CIBB), University of Coimbra, 3004-504 Coimbra, Portugal; 10 Clinical Academic Center of Coimbra (CACC), 3004-504 Coimbra, Portugal; 11 ESTESC-Coimbra Health School, Pharmacy, Polytechnic Institute of Coimbra, 3046-854 Coimbra, Portugal

**Keywords:** 3,4-methylenedioxypyrovalerone, methamphetamine, behavior, glia

## Abstract

3,4-Methylenedioxypyrovalerone (MDPV), a widely available synthetic cathinone, is a popular substitute for classical controlled drugs of abuse, such as methamphetamine (METH). Although MDPV poses public health risks, its neuropharmacological profile remains poorly explored. This study aimed to provide evidence on that direction. Accordingly, C57BL/6J mice were exposed to a binge MDPV or METH regimen (four intraperitoneal injections every 2 h, 10 mg/kg). Locomotor, exploratory, and emotional behavior, in addition to striatal neurotoxicity and glial signature, were assessed within 18–24 h, a known time-window encompassing classical amphetamine dopaminergic neurotoxicity. MDPV resulted in unchanged locomotor activity (open field test) and emotional behavior (elevated plus maze, splash test, tail suspension test). Additionally, striatal TH (METH neurotoxicity hallmark), Iba-1 (microglia), GFAP (astrocyte), RAGE, and TLR2/4/7 (immune modulators) protein densities remained unchanged after MDPV-exposure. Expectedly, and in sheer contrast with MDPV, METH resulted in decrease general locomotor activity paralleled by a significant striatal TH depletion, astrogliosis, and microglia arborization alterations (Sholl analysis). This comparative study newly highlights that binge MDPV-exposure comes without evident behavioral, neurochemical, and glial changes at a time-point where METH-induced striatal neurotoxicity is clearly evident. Nevertheless, neuropharmacological MDPV signature needs further profiling at different time-points, regimens, and brain regions.

## 1. Introduction

Over the last decade, the illicit drug market has become increasingly complex and dynamic with the speed of appearance of new psychoactive substances (NPSs) [1]. NPSs, also known as “synthetic drugs” or “legal highs”, are typically sold via Internet as substitutes for controlled classical drugs of abuse (e.g., opiates, cocaine, and methamphetamine (METH)) [2,3,4,5,6,7]. Synthetic cathinones, more commonly known as “bath salts”, were identified as the main psychoactive compound of products commercialized as “plant feeders” [4] and dominate NPS seizures in European Union (EU, 36%) [1]. These are synthetic derivations of the naturally occurring stimulant cathinone found in the khat plant (amphetamine structural analogue) [2,8]. They may be either N-alkylated or the nitrogen atom is part of a pyrrolidine ring, as is the case of 3,4-Methylenedioxypyrovalerone (MDPV). Synthetic cathinones have also a phenyl ring, which is an important site for chemical modifications. MDPV is the main active ingredient reported and recently added to the list of NPSs monitored by the European Monitoring Centre for Drugs and Drug Addiction (EMCDDA) and Europol [9]. MDPV is considered to have increased potency and abuse potential [3,7]. The clinical effects usually resemble cocaine/amphetamine sympathomimetic syndrome, ranging from altered mental status (e.g., hallucinations, paranoia) to behavioral (e.g., enhanced psychomotor capacity, violent behaviors) and toxic (e.g., acute kidney and liver failure, tachycardia severe fatal cases) consequences [2,3,5]. Similar to other psychostimulants, MDPV targets central nervous system (CNS) plasma membrane transporters for dopamine (DAT), norepinephrine (NET), and serotonin (5-HTT). With relatively poor affinity for 5-HTT, it is a very potent and selective blocker of both DAT (10–50 fold more than cocaine [7,10]) and NET [2,7,11]. MDPV high lipophilicity may be held responsible for its enhanced blood–brain barrier (BBB) permeability [2,3] and drug brain levels [5], hence contributing to its potency at DAT and, therefore, to the dose-dependent increase of extracellular dopamine (DA) in the nucleus accumbens (NAc), often associated with locomotor and rewarding effects [7,12,13]. MDPV has been mostly studied for its psychostimulant features with abuse potential [6], namely the locomotor activity [12,13,14,15,16,17,18,19,20], self-administration behavior [14,19,21,22], conditioned place preference [20,23], and drug discrimination [14,17,24] parameters. Undoubtedly, this synthetic cathinone acts as a potent CNS stimulant, which raises obvious concerns about its potential neurotoxic effects, and possibly amphetamine-like effects, due to their structural similarity. MDPV impact on the vesicular/plasma transporters and the synaptic levels of monoamines, thermoregulation, oxidative stress, and cytotoxicity may represent an important mechanism of action [25]. However, data on the underlying mechanisms of MDPV induced neurotoxicity is not abundant [4,22,26,27] and mostly circumscribed to in vitro studies [28,29,30,31,32,33,34], hampering proper risk characterization and adverse effect management [2,5,25]. Indeed, the scarce data available suggests that synthetic cathinones may exert differential neurotoxic properties on monoaminergic neurons and elicit more complex responses than non-keto amphetamines (e.g., METH) [25]. Contrastingly, a great deal of attention has been devoted to the aspects underlying METH-induced neurotoxicity. For example, METH is well-known for inducing striatal DA nerve terminal damage (e.g., long-term DA, tyrosine hydroxylase (TH) depletion, DAT inactivation, and reduction in function of vesicle monoamine transporter (VMAT)) and degeneration of fine unmyelinated axons and apoptosis [10,26,35]. Additionally, METH induced neuroinflammation has been widely reported either in vitro [36,37,38] or in vivo [35,36,37,39], seemingly mediated by glial activation [35]. Although neuroimmune modulation has been claimed responsible [25,35,40], some authors have recently stood in contrast to those claims of excessive and detrimental neuroinflammation believed to contribute and exacerbate METH neurotoxicity [41]. Overall, both MDPV and METH abuse liability and neurotoxic potential need to be further unraveled.

Accordingly, new avenues are being explored. There is a growing body of evidence showing that a group of pattern recognition receptors (PRRs) that detect and respond to exogenous pathogen associated molecular patterns (PAMPs) and endogenous danger associated molecular patterns (DAMPs), the toll-like receptors (TLRs), may be implicated in psychostimulant induced innate neuroimmune responses [36,40,42,43]. TLR4 signaling seems to be involved in drug reward-associated behaviors and proinflammatory responses, contributing to the development of drug addiction [36,43]. Coherently, the TLR4/NFκB signaling pathway has been recently and increasingly reported to be associated with METH induced activation of glial cells in hippocampus [44], increased DA in the NAc shell [36], enhanced cytokine expression in the cortex [37], and reduced pro-inflammatory mediators in microglia-like cells upon LPS stimulation [38]. Additionally, the receptor for advanced glycation endproducts (RAGE), described to recognize glycosylated proteins and lipids in the forms of advanced glycation endproducts (AGEs), also detecting DAMPs [42], seems to play a pivotal role in synaptic function, glial response, and inflammation homeostasis when upregulated [45]. In fact, our group previously showed that striatum dopaminergic neurotoxicity was associated with increased RAGE transcription and total protein levels [46]. Therefore, it is also envisaged as a potential player on striatal METH neurotoxicity [25,40], an intriguingly overlooked setting. Our group was pioneer in reporting preserved striatal RAGE levels, three days after a single neurotoxic METH dose [47], and further studies are needed to deepen knowledge on different METH regimens, doses, and time-points. Overall, CNS players of neuroinflammation in METH induced neurotoxicity are still being revealed, and largely unknown following MDPV-exposure [13]. This poor pharmacological characterization of MDPV poses a high risk for public health, evident from the reported NPS use intoxications and fatalities [2,3,4,25].

**Figure 1:**
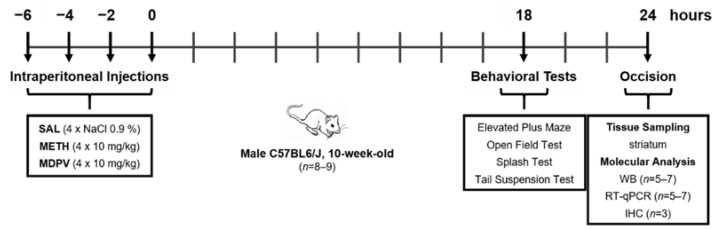
Experimental design. Time course of saline (SAL), methamphetamine-HCl (METH), and 3,4-methylenedioxypyrovalerone-HCl (MDPV) injections, behavioral tests, occision, and tissue sampling.

Therefore, the present work aimed to provide a novel and integrative characterization of the fundamental impact of MDPV on neurotoxicity, exploring classical neuronal and glial markers in addition to putative innate immune modulators and emotional behavior. An MDPV/METH binge paradigm, comprising four intraperitoneal injections (10 mg/kg) every 2 h (Figure 1), was used as an acute model for binge/intoxication stage of addiction [48]. METH was meant to be used both as a comparison measure (same monoaminergic selectivity) and also to deepen current knowledge on METH neurotoxicity. Adult C57BL/6J mice locomotor (open field test (OFT)) and emotional behavior (elevated plus maze (EPM), splash test (ST) and tail suspension test (TST)) were assessed 18–24 h after drug. For the neurotoxic profile, striatal neuron (TH) and glial activity (glial fibrillary acidic protein (GFAP) and ionized calcium binding adaptor molecule (Iba1)) and putative innate immune players (TLRs and RAGE) were evaluated 24 h following MDPV and METH-exposure. Our results show, for the first time, that a binge MDPV-exposure comes without evident behavioral and glial changes, when METH-induced striatal neurotoxicity (striatal TH depletion, astrogliosis, and microglia arborization alterations) is already present. No evidence of immune dysregulation regarding RAGE and TLRs (TLR2, TLR4, and TL7) expression was found. Nevertheless, the characterization of neuropharmacological MDPV signature is critical for understanding its neurotoxic potential at different time-points, doses, and regimens.

## 2. Results

### 2.1. MPDV and METH on Locomotor and Exploratory Activity

**Figure 2:**
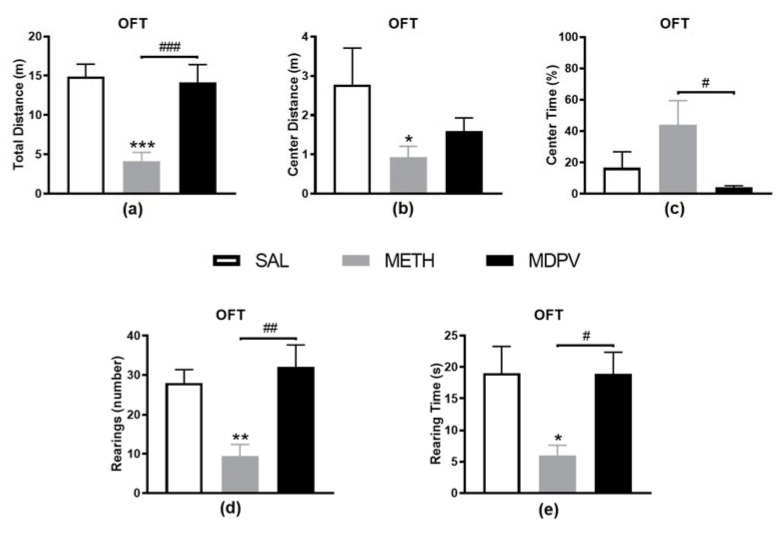
Effect of METH and MDPV binge paradigms on mice behavior in the open field test (OFT). (**a**) Total distance travelled (m); (**b**) center distance travelled (m); (**c**) time spent in center (%); (**d**) number of rearings; and (**e**) rearing time(s). Data are represented as mean ± SEM (*n* = 8–9). Statistical comparisons for total distance traveled and rearing time were made using the one-way ANOVA followed by Tukey’s multiple comparison test and for the other parameters using the Kruskal–Wallis test followed by Dunn’s multiple comparison test (* denotes differences between SAL and METH or MDPV and ^#^ denotes differences between METH and MDPV. * or ^#^, *p* < 0.05; ** or ^##^, *p* < 0.01; *** or ^###^, *p* < 0.001).

In this study, animals exposed to METH showed an overall hypolocomotion compared to SAL, illustrated by the significant decrease in total (Figure 2a, *p* < 0.001) and center (Figure 2b, *p* < 0.05) distance traveled. Additionally, vertical activity levels were also decreased in METH compared to SAL mice for both rearing event (Figure 2c, *p* < 0.01) and time (Figure 2d, *p* < 0.05) measures, suggesting that METH-exposed mice were less active than the SAL. MDPV exposed mice exhibited an OFT performance similar to the SAL. Nevertheless, we might highlight the decreased center walking distance (Figure 2b, 43% decrease) and time (Figure 2c, 75% decrease), although not statistically significant. Moreover, MDPV and METH exposed mice differed in both horizontal (Figure 2a,c, *p* < 0.001 and *p* < 0.05) and vertical (Figure 2d,e, *p* < 0.01 and *p* < 0.05) locomotor activities. Overall, the variations observed between MDPV and METH were similar to those observed between METH and SAL, suggesting that MDPV may induce a normal locomotor and exploratory behavior 24 h after four injections of 10 mg/kg, resembling the SAL group.

### 2.2. MPDV and METH on Emotional Activity

**Figure 3:**
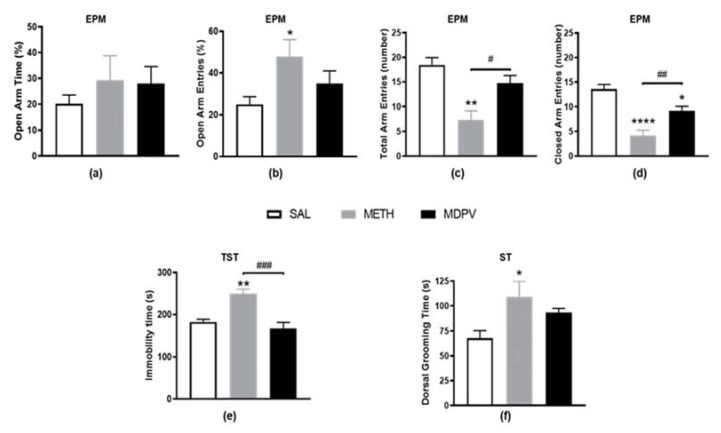
Effect of METH and MDPV binge paradigms on mice emotional behavior in elevated plus maze (EPM), tail suspension (TST), and splash tests (ST). In EPM test, the following parameters were analyzed: (**a**) time spent in open arms (%); (**b**) entries in open arms (%); (**c**) number of total arm entries; and (**d**) number of closed arm entries; (**e**) immobility time during the TST; and (**f**) dorsal grooming time during the ST. Data are represented as mean ± SEM (*n* = 8–9). Statistical comparisons for number of closed arm entries and dorsal grooming time were made using the one-way ANOVA followed by Tukey’s multiple comparison test and for the other parameters using the Kruskal–Wallis test followed by Dunn’s multiple comparison test (* denotes differences between SAL and METH or MDPV and ^#^ denotes differences between METH and MDPV; * or ^#^, *p* < 0.05; ** or ^##^, *p* < 0.01; ^###^, *p* < 0.001; ****, *p* < 0.0001).

The EPM test was performed to assess stress-induced mice anxiety-like behavior (Figure 3). This test is very sensitive to treatments that produce disinhibition and stress, and it is regarded as a classic animal model of “emotionality” [48]. In this study, and consistently with the OFT results, METH exposed mice also showed significant hypolocomotion during the EPM, evidenced by decreased measures of total locomotion on the maze (total and closed arm entries, Figure 3c,d, *p* < 0.01 and *p* < 0.0001). Although the percentage of open arm time was not affected after METH-exposure (Figure 3a), an increased percentage of open arm entries was observed compared to the SAL group (Figure 3b, *p* < 0.05). The locomotor alterations seen in this apparatus hinder any assumption regarding these behavior alterations in the open arms, which probably reflect diminished total arm entries compared to SAL (Figure 3c). Nevertheless, the increased immobility time in the TST (Figure 3e, *p* < 0.01), and the increased dorsal grooming time in the ST (Figure 3f, *p* < 0.05) of METH exposed mice are suggestive of an emotional disturbance and stress-like behavior. On the other hand, the emotional behavior of MDPV exposed seemed to be globally unaffected and similar to SAL. Even so, although no differences were observed on open arm entries and time (Figure 3a,b) and total arm entries (Figure 3c), a reduction in the number of closed arm entries was seen compared to SAL (Figure 3d, *p* < 0.05). This, for the same reason clarified above, might explain the trend observed towards increased percentage of open arm entries (Figure 3b, 40% increase). In sharp contrast with METH, MDPV showed no evidence of depressive-like behavior in either TST (Figure 3e, *p* < 0.01) or ST (Figure 3f, *p* < 0.05). However, a tendency to increased dorsal grooming time was observed compared to the SAL condition (Figure 3f, 37% increase). Overall, METH, but not MDPV, seems to significantly affect mice emotional behavior.

### 2.3. MPDV and METH on Striatal Dopaminergic Terminals 

**Figure 4:**
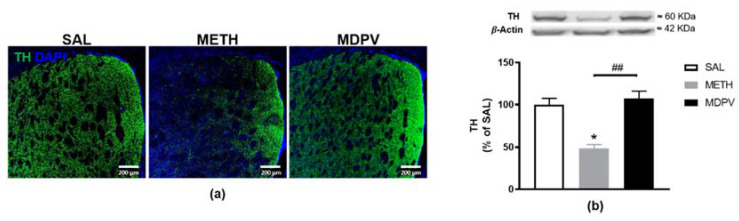
Effect of METH and MDPV binge paradigm on mice striatum TH protein density. (**a**) Representative confocal images of striatal sections, co-labelled with anti-TH antibody (green) and DAPI (blue) (scale bar: 200 μm; *n* = 3); (**b**) representative western blot and quantification of striatal TH protein density. Results were normalized with β-actin and expressed as mean % of saline ± S.E.M (*n* = 5–7). Statistical comparisons were made using the Kruskal–Wallis test followed by Dunn’s multiple comparison test (* denotes differences between SAL and METH or MDPV and ^#^ denotes differences between METH and MDPV. *, *p* < 0.05; ^##^, *p* < 0.01).

TH is the rate-limiting enzyme in DA synthesis and is the golden marker for dopaminergic terminals in striatum. Importantly, depletion of striatal TH is a hallmark of METH-induced neurotoxicity [35,49]. Accordingly, to assess METH and MDPV-induced damage to the dopaminergic terminals in the striatum, we analyzed the striatal TH expression. The immunostaining results revealed significant TH loss in the striatum of METH exposed mice (Figure 4a), further confirmed and quantified as a 50% depletion by western blot analysis (Figure 4b, *p* < 0.05). On the other hand, as illustrated in Figure 4, MDPV failed to induce changes in TH levels, exhibiting similar immunoreactivity and protein density as the SAL group and significantly higher striatal TH levels than METH (Figure 4b, *p* < 0.01). Results suggest METH induced neurotoxicity, consistent with our previous studies using a single neurotoxic METH dose [47,50], but not MDPV.

### 2.4. MPDV and METH on Striatal Glial Reactivity

**Figure 5:**
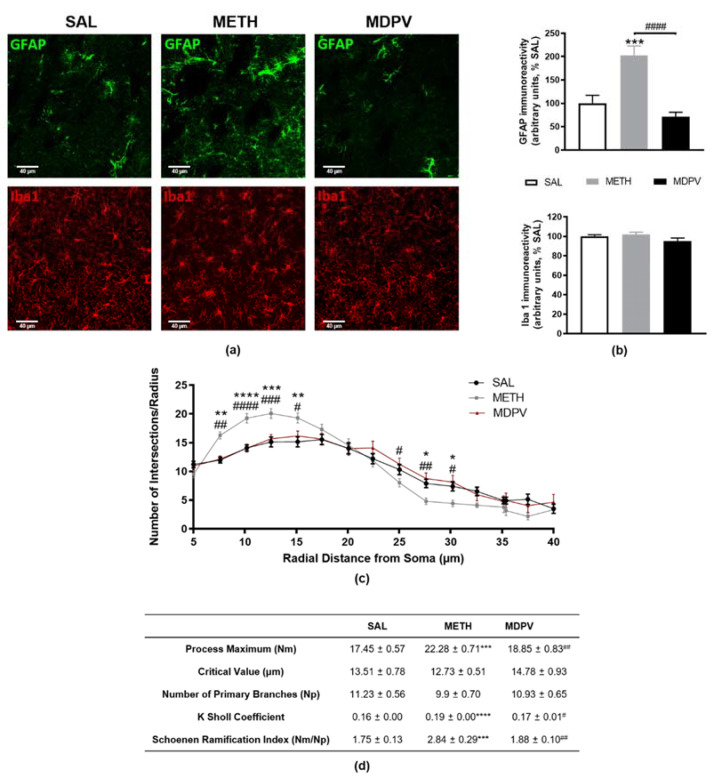
Effect of METH and MDPV binge paradigm on mice glial status. (**a**) Representative confocal images of striatal sections co-labelled with anti-GFAP (green) and Iba-1 (red) antibody (scale bar: 40 μm; *n* = 3 animals); (**b**) GFAP and Iba-1 immunoreactivity quantification; (**c**) Sholl plot and (**d**) morphological parameters of microglia cells (40 cells/group). Results were expressed as mean% of saline ± S.E.M (*n* = 3 animals). Statistical comparisons were made using the one-way ANOVA followed by Tukey’s multiple comparison test (* denotes differences between SAL and METH or MDPV and ^#^ denotes differences between METH and MDPV. * or #, *p* < 0.05; ** or ##, *p* < 0.01; *** or ###, *p* < 0.001; **** or ^####^, *p* < 0.0001).

To unravel the effect and possible differences between MDPV and METH on glial reactivity, a possible contributor to psychostimulant induced neurotoxicity, we evaluated astrocytic GFAP and microglial Iba-1 markers in striatal coronal sections (Figure 5). METH-exposure induced twice more fluorescence signal of GFAP-positive astrocytes than the SAL condition (Figure 5a,b, *p* < 0.001), suggesting astrogliosis. Once again, a clear difference was found between METH and MDPV regarding striatal GFAP (Figure 5a,b, *p* < 0.0001). On the other hand, no alterations were observed between groups regarding microglial Iba-1 marker for either MDPV or METH. Nevertheless, we were intrigued by the apparent misshape found in microglia in METH-treated mice (Iba-1 seems to exhibit a higher concentration of signal in microglia somas than in surrounding ramifications, Figure 5a). Accordingly, microglia activation is translated in dynamic changes over time, starting from retraction of the processes and further enlargement of the soma [51]. Therefore, a Sholl analysis was performed to explore if at the present time-point (24 h), there were subtle changes in microglia morphology between groups. Figure 5c,d illustrate the Sholl plot and the morphological parameters of microglia cells (40 cells/group). In fact, the obtained results confirmed our hypothesis, showing an early state of microglial reactivity in METH-treated mice. The number of processes along the cell clearly had higher branching near the soma, being most significant at a radial distance of 10 μm (Figure 5c, *p* < 0.0001), and exhibiting a tendency to decrease at longer radial distance (significant at 27.5 and 30 μm, *p* < 0.05). This seemed to happen without changing the number of primary branches (Np) (Figure 5d). The increased Sholl coefficient (K) also mirrors the decrease in process density along the microglia (Figure 5d, *p* < 0.0001), and the increased Schoenen ramification index (Figure 5d, *p* < 0.01) indicates microglia arborization. Sholl analysis did not show any difference between MDPV and SAL group (Figure 5c), in line with the Iba-1 immunostaining quantifications (Figure 5b). Once again, these results are consistent with METH induced neurotoxicity, astrogliosis, and unique microglial alterations not observed for MDPV at the present time-point.

### 2.5. MPDV and METH on Immune Modulators RAGE and TLR

**Figure 6:**
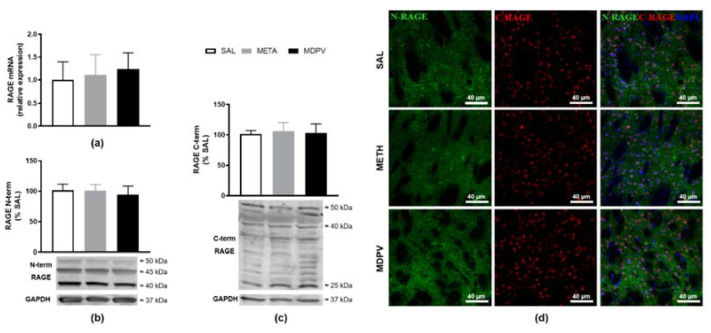
Effect of METH and MDPV binge paradigm on mice striatal RAGE mRNA relative expression, protein density and sub-cellular distribution. (**a**) RAGE mRNA expression, expressed as mean % NRQ of saline ± S.E.M. (*n* = 5–7); (**b**,**c**) Representative western blot and quantification of striatal RAGE proteins with anti-RAGE N-terminal and anti-RAGE C-terminal antibodies, respectively. Results were normalized with GAPDH and expressed as mean % of saline ± S.E.M. (*n* = 5–7); statistical comparisons were made using the Kruskal–Wallis test followed by Dunn’s multiple comparison test; (**d**) representative confocal images of striatal sections, co-labelled with anti-RAGE N-terminal antibody (green) and anti-RAGE C-terminal antibody (red). Sections were counterstained with DAPI (blue) for nuclei visualization (scale bar: 40 μm, *n* = 3).

The suggestion of astrogliosis and early microglia activation induced by METH-exposure, prompted us to further analyze other immune parameters that might be associated with this glial activation. PPRs have been discussed as involved in psychostimulant neurotoxicity, and our group already linked striatum dopaminergic neurotoxicity with increased RAGE [46]. Therefore, as a complementary approach, we evaluated the impact of these dopaminergic toxins on the overall isoform-level of RAGE (Figure 6). Binge-like METH or MDPV regimen did not impact RAGE mRNA levels for 24 h following the last drug administration (Figure 6a). As previously described [46], protein quantification through western blot allowed us to distinguish RAGE-isoforms, using antibodies specific to each terminal domain. Using an antibody raised against the N-terminal, we were not able to detect any differences in RAGE immunoreactivity for monomeric monomeric flRAGE (50 kDa) or inhibitory variants (45/40 kDa) (Figure 6b). An anti-C-terminal RAGE antibody also did not show changes in immunoreactivity for flRAGE (50 kDa), pre-glycosylated flRAGE/N-truncated isoforms (40 kDa), or proteolytic product of RAGE (25 kDa) (Figure 6c). Accordingly, immunolabeled brain sections of each condition did not show differences in RAGE sub-cellular distribution (C-terminal RAGE-nuclear staining and N-terminal RAGE-cytosolic staining) between treatments and SAL (Figure 6d). Additionally, the other PRRs evaluated, TLR 2, 4, or 7 did not seem to have different striatal protein densities for the METH or MDPV compared to the SAL group (Table 1).

## 3. Discussion

The present work aimed to provide additional insight into the neuropharmacological mechanisms of MDPV and METH use and abuse. The main study novelty was the assessment of both emotional behavior and neurotoxicity related glial reactivity and neuroimmune modulation, poorly elucidated after both MDPV- and METH-exposure. We decided to use a model for the binge/intoxication stage of addiction, mimicking MDPV and METH patterns of recreational use [48]. Particularly, this MDPV/METH binge paradigm, comprising four intraperitoneal injections with a 2-h interval between each injection (Figure 1), is a gold standard acute model to study early neurochemical imprint of METH neurotoxicity, resulting in extensive DA nerve ending damage and associated behavioral changes [52,53,54]. Additionally, it falls well within the dose range used in similar MDPV published studies [4,12,17,19,26]. Overall, the obtained results indicated minor MDPV alterations at this time-point. On the other side, results were clearer on the METH induced neurotoxicity, triggering evident hypolocomotion accompanied by striatal TH depletion, astrogliosis, and unique microglia alterations. We must clarify that no significant differences were observed in terms of food and water intake, or in body weight along the 24 h after exposure to both MDPV and METH.

Regarding the behavioral analysis, the significant hypolocomotion found in METH mice and the absent alterations in the locomotor profile of MDPV mice, 24 h after the last dose, are in agreement with the results reported by others for these psychostimulants. In fact, although MDPV single [12,14,15,16,18,20] and binge/intermittent regimens [20] immediately elicited locomotor activation in rodents, studies conducted over time found a transient effect peaking within 0.5–1 h and decreasing to basal levels over 5–6 h [13,17,18]. Consistently, MDPV displays rapid pharmacokinetics, with peak plasma concentrations achieved at 10–20 min and declining quickly thereafter [11]. In addition, MDPV reached the brain 5 min after a subcutaneous administration and peaked 20–25 min later, and the striatum elimination half-life occurred 61 min later, coincident with decreased psychostimulant effect [18]. This immediate effect on locomotion seems to occur through a DA dependent mechanism, as pretreatment with both selective [13] and non-selective [18] DA receptor antagonists reversed those effects. Moreover, MDPV dose-dependent effects were also considered by several authors [12,13,14,16,17,18,55]. While some saw a dose-dependent increase in increased horizontal and vertical activity, mostly observed within the first 1–2 h [13,18], others have found no dose-dependent effect [12,17] or even correlated increasing doses with decreased wheel activity [16]. Deeply contrasting to MDPV, METH caused significant impairment in horizontal and vertical locomotor activity, corroborating our previous findings after a single high METH dose (30 mg/kg) [47,56] and others following a similar METH binge regimen (4 × 10 mg/kg) [52,54,57]. That hypolocomotion was also confirmed during the EPM test and, therefore, did not enable us to draw assertive conclusions regarding mice anxiety status. A depressive-like behavior could be suggested by the TST, and a stress-induced self-care disturbance was observed during the ST. Indeed, long-lasting depressive-like behavior was already reported by us [50] and others [58,59] following diverse METH regimens in rodents, and it is a commonly experienced withdrawal symptom in METH users. Nevertheless, we cannot exclude that this behavioral alteration may be, at least partially, attributed to the magnitude of the observed motor deficits. Regarding stress induced self-care dysfunction measures inferred during the ST, one might expect decreased grooming activity as a reflection form of motivational behavior, paralleled with some symptoms of depressive-like behavior such as apathy or an attenuation of sucrose preference [20]. On the other hand, it may represent a typical displacement behavior, often increased in animals under conditions of stress and, thus, viewed as an indirect measure of an anxiogenic response (to psychotropic drugs for instance) [60]. Indeed, anxiety is a negative emotional state critical to survival, but persistent and exaggerated apprehension causes substantial morbidity [61]. Overall, our data further show that METH animals have locomotor and emotional impairments. The effects of MDPV administration on negative emotional states, namely on anxiety- (EPM) [20,62], hedonic- (sucrose preference test (SPT)) [20], and depressive-like behavior (forced swim test (FST)) [63], have been poorly investigated to date. This is the first study evaluating the effect of a binge MDPV-exposure on ST and TST measures. This cathinone did not cause an anxiety- and depressive-like behavior at this particular time-point. However, others using different dosing paradigms, time-points and species (rat vs. mice) showed significant emotional alterations. For example, while anxiolytic-like effects (increased number of entries and time in EPM open arms) were reported 1 h after a binge MDPV regimen (3 × 3 mg/kg, for three days) [20], no influence was seen on the same measures at two or 21 days [62]. On the other side, an anxiogenic-like behavior was reported after longer MDPV administration schedules (3 × 1 mg/kg, for 10 days) [63]. These authors also reported alterations suggestive of a depressive-like behavior (FST increased immobility), contrasting with our TST results. Interestingly, there was a 37% increase in dorsal grooming time for MDPV (although not reaching statistical significance), mimicking the METH-induced stress and disturbed self-grooming (not apathy) [60]. Our novel data outline a new behavioral profile for MDPV, 18 h after a high-dose binge regimen, where the stimulant effect is already absent, leaving behind apparent traces of emotional impairment as gauged by a reduction in time and distance in the center of the OFT apparatus, which is seemingly a stressful environment to animals [64]. For METH, the present approach states the importance of having complete behavioral batteries to avoid hastening conclusions, since certain behaviors may be shaded by others. 

MDPV- and METH-exposure may induce important modifications on CNS. Therefore, the second aim of our study was to examine the effects on striatal molecular and cellular outcomes. The locomotor impairment observed has been often associated with METH induced early striatal neurotoxicity, characterized by depletion of dopaminergic–terminal markers (TH and DAT) and glial reactivity [39,47,50,65,66,67,68,69,70]. Specifically, the nearly 50% depletion of TH-immunoreactive fibers presently observed meet the published studies using similar drug-regimens, suggesting TH as an established initial and sensitive marker to evaluate METH-striatal toxicity. Furthermore, our results also revealed that the dopaminergic insult was accompanied by increased GFAP-immunostaining of slightly enlarged astrocytes, suggesting an early stage of astrocytic activation. Consistently, similar drug-regimens showed a peak of astrogliosis over three days [47,54,67,68]. Contrary to our expectations, no apparent activation of microglia (Iba-1 immunostaining) was found. Nevertheless, as Iba-1 staining was seemingly more evident around microglia somas when compared to SAL, we considered a Sholl analysis, as a highly sensitive approach to further analyze microglia morphology and complexity. Indeed, the results revealed morphological changes in microglia after METH-exposure, showing higher branching near the soma, together with a decrease in the number of processes along microglial cells. These data may represent early stages of microglial activation, before acquiring an amoeboid shape of the soma with retracted and shortened processes. In fact, microgliosis was already described 24 h after METH-exposure [68]. 

On the other hand, little is known about the possible neurotoxicity produced by MDPV-exposure. As both toxins share selectivity for the dopaminergic system [25], we hypothesized that the MDPV binge regimen could also induce striatal dopaminergic toxicity associated with TH depletion within the first 24 h. Our work is pioneer in the assessment of striatal MDPV-induced toxicity with an administration protocol that replicates a METH binging paradigm that consistently causes striatal neurotoxicity. In agreement with the normal locomotor activity observed, no alterations were seen in striatal TH, GFAP, or Iba-1 protein semi-quantification or immunostaining. Therefore, no evidence of the classical markers of METH-neurotoxicity was found. From the five published studies on MDPV induced striatal toxicity (DA, DAT, TH, and GFAP levels) [22,26,27,62,71], three support our results, reporting no alterations 48 h after a high [26] or low [27] dose short binge regimen (4 × 30 or 1 mg/kg, every 2 h) or 24 h after a single MDPV administration (2.5 or 5 mg/kg) [71]. On the other hand, some alterations were reported after longer binge regimens (2 × 1.5 mg/kg, for seven days) [62] or single MDPV doses [22,71]. Duart-Castells et al. reported unchanged DAT, but increased TH and D1 DA receptor (DRD1) 24 h after the last dose [62]. Increased DAT activity was reported as early as 1 h post MDPV-exposure [22]. This is in line with the observations of Lopez-Arnau et al. that showed a rapid and reversible functional upregulation of DAT in synaptosomes (increasing 1–3 h after 1.5 mg/kg MDPV-exposure, but not 16 h later), more powerfully and lasting than cocaine [72]. These observations suggest that MDPV triggers an early and transient DAT effect. The authors argued that this effect takes place at the nerve terminal, and may be a response to the intense DAT blockade exerted by MDPV [72]. In contrast to both Lopez-Arnau et al. [72] and Colon-Perez et al. [71], Magee et al. did not find evidence of altered DAT surface expression [71]. Additionally, repeated doses of MDPV developed tolerance to that DAT upregulation, which was reduced 24 h after five days of MDPV daily administrations [72]. Moreover, MDPV was shown to completely protect against METH neurotoxicity, by blocking DAT-mediated transport [10], preventing METH uptake and striatum DA nerve ending characteristic METH induced damage hallmarks (DAT and TH reduction and GFAP upregulation) [26]. The following MDPV pharmacological properties may account for its neurochemical profile: 1—MDPV is a DAT blocker (not a substrate [73]) with a strong molecular affinity and, therefore, is not internalized to the cytoplasm as quickly as METH [10]; 2—MDPV high lipophilicity and presumed transendothelial active transport [10] contributes to its short half-life (≈1–2 h, in men) [18,74] compared to METH (≈10 h, in men) [75]; 3—MDPV potential dual action on DAT (one to inhibit and a second to enhance DAT function) might contribute to its highly reinforcing properties while also mitigating persistent dopaminergic deficits due to aberrant DA transport [22]. Additionally, Araújo et al. performed in vivo toxicometabolomics following a MDPV binge regimen similar to the one we used and consistently reported minor changes in the brain [76]. The authors found altered 3-hydroxybutyric acid levels, which may reflect the activation of a neurotoxic pathway, but the increase in metabolites with neuroprotective properties seemed to counteract this change. Overall, previous results support the absence of striatal dopaminergic neurotoxic effects seen with MDPV, in contrast with METH-induced striatal profile. 

Lastly, we hypothesized that putative MDPV and METH neurotoxicity would be mediated by CNS players of innate immunity and neuroinflammation. In fact, the observed METH effects on glial cell activity (both astrocytes and microglia) may be intrinsically linked with activation of inflammatory processes underlying neuronal damage [13]. Thus, alterations in immune cell function can be held responsible for METH neuroinflammation and addictive effects [40], which are strongly associated with neuronal impairment and generalized brain dysfunction usually present in drug abusers [77]. MDPV is also expected to interfere with inflammatory pathways [78]. Nevertheless, neuroinflammation markers have not been documented after MDPV in vivo administration. Accordingly, we aimed to characterize if PRR innate immune modulators, particularly the TLRs and RAGE may be altered, as both have been implied in microglia induced neurotoxicity and neuroinflammation in drug addiction [25,36,40,42,43,45,46,79]. Unique attention has been given to TLR4 signaling: this is seemingly associated with METH induced activation of glial cells in hippocampus [44], increased DA in the NAc shell [36], enhanced cytokine expression in the cortex [37], and reduced pro-inflammatory mediators in microglia-like cells upon LPS stimulation [38]. However, our results dismiss the assumption that either striatal RAGE or TLR2, TLR4, and TLR7 could be directly involved in the observed glial activation. These results are in line with our previous findings that showed unchanged striatal RAGE density after a single high-dose METH-exposure [47]. These innate immune modulators were also unchanged following MDPV-exposure. This is in line with the absent neurotoxicity reported for this particular MDPV protocol, dose, time-point, and brain region. Overall, the link between neuroinflammatory pathways and psychostimulant neurotoxic effects needs to be further explored.

The present study has some limitations that should be mentioned. First, only male mice were used and, therefore, results cannot be generalized for both sexes. Female mice were not used to avoid estrous cycle and hormonal impact on data variability, mostly on behavioral performance. Additionally, we cannot discard that MDPV impact in some neuronal signaling pathways, and glial activation might be sex-dependent. Second, we must highlight that using only a single time-point behavioral and neurochemical analysis is a major pitfall. Third, one should not discard that possible damages in other brain regions beyond striatum (including hippocampus and prefrontal cortex, known targets for psychostimulants) are in place at this time-point. Therefore, further research is warranted to highlight the complex CNS response to MDPV, namely including both sexes, different time-points, and brain regions. Although the present study contributes to a thorough characterization of an acute MDPV binge exposure in vivo, its translation to the clinics should be done with caution, for all the limitations mentioned, and also because of the complexity of drug abuse social and ethical issues. However, minor MDPV behavioral alterations seen with this acute paradigm suggest that the harm-reduction guidance at the clinical level should be done in accordance with that available for other stimulant and cathinone intoxication settings

## 4. Materials and Methods

### 4.1. Animals

Male adult C57BL/6J mice (10-week-old, 21–27 g, Charles River Laboratories, Barcelona, Spain) were housed, three–four per cage, and maintained under controlled environmental conditions (12/12 h light/dark cycle at 23 ± 1 °C, with food and water ad libitum). All experiments were approved by the Institutional Animal Care and Use Committee from Faculty of Medicine, University of Coimbra (ORBEA_251_2020/05032020), following the European Community directive (2010/63/EU) and Portuguese law for care and use of experimental animals (DL no. 113/2013), and in compliance with Animal Research: Reporting of In Vivo Experiments (ARRIVE) guidelines. Animals were randomly allocated to three groups, and the total body weight was balanced before starting the experiment: saline (SAL), METH, and MDPV (*n* = 8–9). All efforts were made to minimize animal suffering and to reduce the number of animals used.

### 4.2. Drugs and Chemicals

We were issued permission by INFARMED (Portuguese National Authority of Medicines and Healths Products) to import methamphetamine-HCl (METH) from Sigma-Aldrich (St. Louis, MO, USA). 3,4-methylenedioxypyrovalerone-HCl (MDPV) was purchased online from the Sensearomatic website (http://sensearomatic.net, accessed on 4 February 2016, currently unavailable). The other chemicals used were from Sigma-Aldrich and Merck AG (Darmstadt, Germany).

### 4.3. Drug Administration

All mice were subjected to a binge-like regimen comprising four intraperitoneal injections with 2 h interval: (i) SAL (4 × 0.9 % NaCl), (ii) METH (4 × 10 mg/kg METH), and (iii) MDPV (4 × 10 mg/kg MDPV). This binge-like treatment regimen was chosen because it results in extensive DA nerve ending damage and astrogliosis at 12–24 h, when used to inject substituted amphetamines [67]. Additionally, the dose of 40 mg/kg of METH falls approximately in the range of a typical human single-use overdose via intraspecies scaling [48]. We decided to test MDPV in the same dose for direct comparison between both drugs tested and also because it falls well within the dose range used in similar MDPV in vivo studies [12,17,19,26]. Finally, all animals survived this dosing regimen, and none showed convulsions or weight reductions.

### 4.4. Behavioral Analysis

A battery of behavioral tests was conducted in three independent cohorts from 18 to 24 h after the last drug administration, in the following order: elevated plus maze test (EPM), open field test (OFT), splash test (ST), and tail suspension test (TST). Behavioral tests were carried out between 9 am and 1 pm in a sound-attenuated room under dim light (10 lx), following 1 h habituation. All apparatuses were cleaned with 10% ethanol between tests, to avoid animal odor and clues. Mice behavior was scored by the same rater and monitored through a video camera. Afterwards, a blind analysis was performed by an external experienced researcher using the ANY Maze video tracking (Stoelting Co., Wood Dale, IL, USA).

#### 4.4.1. Elevated Plus-Maze

The EPM was used to evaluate the anxiety-related behavior in mice. It was performed in a gray acrylic apparatus of four arms placed 55 cm above the floor (LE 848 PANLAB, Barcelona, Spain). The four arms measured 18 cm length and 6 cm wide, and two opposing arms were surrounded by opaque gray walls (15 cm height, closed arms), while the other two arms were devoid of walls (open arms). Each animal was placed in the center of the apparatus, facing an open arm, and its exploratory behavior was measured during 5 min. Time spent in open arms (%), open arm entries (%) (anxiogenic-like behavior), total arm entries, and closed arm entries (number) (locomotor activity) were the evaluated parameters [80].

#### 4.4.2. Open Field Test

Mice were individually placed for 5 min in the center of an open field arena (45 × 45 × 45 cm, light gray acrylic apparatus) to evaluate the impact of METH or MDPV on spontaneous locomotor and exploratory activities as well as on anxiogenic-like behavior. Total distance travelled (m), number of rears, and rearing time (s) as indicators of spontaneous locomotor and exploratory activity, center distance travelled (m), and time in center (%) as indicators of anxiogenic-like behavior were analyzed [64,81].

#### 4.4.3. Splash Test

The ST test was used to evaluate the impact of METH or MDPV on self-care behavior. For this purpose, a 10% sucrose solution was vaporized on the dorsal coat of each mouse, individually, in its home cage. The grooming behavior (dorsal grooming of dirt coat) was recorded for 5 min, as a validated tool to probe stress-induced self-care and motivational behavior [60].

#### 4.4.4. Tail-Suspension Test

The TST is a predictive behavioral test of antidepressant activity and also other manipulations, including pharmacological (e.g., METH and MDPV), that may trigger depressive-like behavior. The test is based on the inability of mice to exhibit escape-directed behavior, when subjected to a short-term stress, thus developing a still posture. Decreased immobility (i.e., increased escape-directed behavior) strongly correlated with antidepressant effects in humans. For that purpose, mice were suspended 50 cm above the floor using adhesive tape placed approximately 1 cm from the tip of the tail, for 6 min. The immobility time, when they hung passively and completely motionless, was further analyzed [50].

### 4.5. Tissue Collection and Processing

Mice were euthanized by decapitation, 2 h after completing the behavioral tests (24 h after METH/MDPV) following ketamine (120 mg/kg; Imalgène) and chlorpromazine (10 mg/kg; Largactil) intraperitoneal anesthesia. For RT-qPCR/WB experiments, striata were dissected on ice and quickly stored at −80 °C (*n* = 5–7). The remaining animals (*n* = 3) were submitted to transcardial perfusion fixation (10 mL of 0.01 M PBS, pH 7.4, followed by 20 mL of 4% PFA in 0.01 M PBS, pH 7.4). Mice brains were removed and post-fixed (4% PFA for 24 h at 4 °C, 20% sucrose in 0.01 M PBS, pH 7.4, for at least 24 h at 4 °C).

### 4.6. RT-qPCR Gene Expression

RT-qPCR protocol was performed as previously described [46]. Briefly, RNA was isolated from striata according to protocol from RecoverAll^TM^ Total Nucleic Acid isolation kit (AM1975, AlfaGene Bioscience Inc., Fairfield, NJ, USA). Total amounts of RNA extracted, RNA integrity (RIN, RNA Integrity Number), and purity (A260/A280) were measured by RNA Nano Chip kit in Agilent 2100 Bioanalyzer (2100 expert software, Agilent Technologies, Walbronn, Germany) and ND-1000 spectrophotometer (NanoDrop Technologies, Wilmington, DE, USA), respectively. RNA was reverse transcribed with Transcriptor Universal cDNA Master (Roche Diagnostics, Mannheim, Germany): 1 μg of total RNA was mixed with a 5× Transcriptor Universal Reaction Buffer and 20× Transcriptor Universal Reverse Transcriptase (Roche Diagnostics) in a total reaction volume of 20 μL. Reactions were carried out in a thermocycler Eppendorf vapo.protect with the following thermal profile: 5 min at 25 °C, 10 min at 55 °C, and 5 min at 85 °C. Gene expression was performed by real time quantitative polymerase chain reaction (RT-qPCR) using LightCycler 480 II system (Roche Diagnostics). RT-qPCR amplification of RAGE and endogenous controls 18SrRNA, YWHAZ, and β-actin used optimized primers from Real time ready catalog assays (Cat. No. 31148, 300236, 307906, and 317883, respectively, Roche Diagnostics) and LightCycler 480 Probes Master 29 (Roche Diagnostics), according to manufacturer’s instructions. The primer mouse sequences used were as follows: RAGE, 5′-GTCAGCATCAGGGTCACAGA-3′ (forward) and 5′-AAGGCCAGGGCTAGCGTA-3′ (reverse); 18SrRNA, 5′-GCAATTATTCCCCATGAACG-3′ (forward) and 5′ GGGACTTAATCAACGCAAGC-3′ (reverse); b-actin, 5′ CTAAGGCCAACCGTGAAAAG-3′ (forward) and 5′-ACCAGAGGCATACAGGGACA-3′ (reverse); Ywhaz, 5′-CTTCCTGCAGCCAGAAGC-3′ (forward) and 5‘-GGGTTTCCTCCAATCACTAGC-3′ (reverse). Non-template control reactions were performed for each gene, in order to assure that there was no unspecific amplification. RT-qPCR results were analyzed with qbase+ software (Biogazelle, Gent, Belgium). The relative expression ratio of each of the target genes was computed based on its real-time PCR efficiencies (E) and the crossing point difference (DCq) for an unknown sample versus a control (EDCq method). Results were obtained in normalized relative quantities and then converted to percentage using control group as reference.

### 4.7. Western Blot

Total striatal protein extracts and western blot (WB) analysis were performed as previously described [46,50]. Briefly, striata was lysed in RIPA buffer with protease (Roche Applied Science, Basel, Switzerland) and phosphatase (Phosphatase Inhibitor Cocktail A sc-45044, Dallas, TX, USA) inhibitors. Supernatant protein concentration was determined using the BCA Protein Assay Koy (Pierce Biotechnology, Rockford, IL, USA). Primary antibodies (Table A1) and alkaline phosphatase-conjugated IgG secondary antibodies were used. Membranes were visualized on Thyphoon FLA 9000 (GE Healthcare, LittleChalfont, UK) and analyzed using Image Quant 5.0 software (Molecular Dynamics, Inc., Sunnyvale, CA, USA). Results were normalized against internal controls b-actin/Glyceraldehyde 3-phosphate dehydrogenase (GAPDH) and then expressed as percentage of control. 

### 4.8. Immunohistochemistry

Striatal coronal sections of 40 µm thickness were collected from cryostat (Leica CM3050S, Nussloch, Germany) in antifreezing solution and used for free-floating immunohistochemistry, as previously described [46]. Slices were incubated with primary antibodies overnight at 4 °C and with the respective secondary antibodies for 2 h at room temperature (Table A1). Nuclei were visualized after 4′,6-diamidino-2-phenylindole (DAPI; 1:5000; D1306, Invitrogen, Carlsbad, CA, USA) staining. Images were acquired from five slices per animal (*n* = 3) using a confocal laser-scanning microscope (LSM 710 Meta, Carl Zeiss Gottingen, Germany). Total fluorescence intensity of GFAP and Iba-1 labelling were quantified in FIJI Software version 2.0. All photograph areas were considered, and three different zones without staining (black) were used for background subtraction. Corrected total GFAP and Iba-1 fluorescence were determined as follows: correct total fluorescence = (integrated intensity) − (area of picture × mean background), and results were expressed as mean of fluorescence intensity (arbitrary units), as previously described [52]. To quantify morphological changes of Iba-1^+^ cells, consecutive Z-stack images were converted to a maximum intensity projection image, thresholded by Fiji Software version 2.0. Using the Image J Sholl plugin [82], we analyzed 40 cells of each experimental condition. For each cell, we removed surrounding processes manually using Fiji Software. Through the line segment tool, we draw a line from the center of each soma to the tip of its longest process, thus providing the maximum process length. Concentric circles were drawn centered on the soma, beginning at 5 μm radii and increasing 2 μm with every circle. We determined the number of intersections made by microglia branching processes with each successive increasing circle to create a Sholl plot. From these data, the maximum number of intersections (Nm, the highest number of intersections regardless the radius value), the critical value at which Nm occurred (Cr), and the number of primary branches (Np, the number of branches that originated from the microglia soma) were determined. From these parameters, the Shoenen ramification index (Nm/Np) was calculated to quantify cell branching density as well as K Sholl coefficient [83]. 

### 4.9. Statistical Analysis

Data are depicted as mean values ± SEM and statistical analyses were performed using GraphPad Prism 6.0 software for Windows (GraphPad Software, La Jolla, CA, USA). Data from behavioral experiments were tested for normality using D’Agostino–Pearson’s test and further compared with the parametric test one-way analysis of variance (ANOVA) with post-hoc Tukey’s multiple comparison test. Non-normal data were compared using the non-parametric Kruskal–Wallis test with post-hoc Dunn’s multiple comparison test. Remaining data were tested for normality using the Kolmogorov–Smirnov test and further compared with the parametric test one-way ANOVA with post-hoc Tukey’s multiple comparison test. The accepted level of significance for the tests was *p* < 0.05, were * denotes differences between SAL and METH or MDPV and ^#^ denotes differences between METH and MDPV groups. * or ^#^, *p* < 0.05; ** or ^##^, *p* < 0.01; *** or ^###^, *p* < 0.001; and **** or ^####^, *p* < 0.0001.

## 5. Conclusions

The binge regimen used for both METH and MDPV enabled comparative conclusions regarding the neuropsychopharmacology of these psychostimulants. METH was able to reproduce previous findings of locomotor and emotional impairment, striatal dopaminergic–terminal damage, and astrogliosis. We additionally provided for the first time a distinct microglial morphological profile using Sholl analysis, which is suggestive of microgliosis. Therefore, we further consolidate METH neurotoxic profile. This study also provides a new and integrative insight on the effect of an acute MDPV binge paradigm on behavior, neurochemical, and glial parameters. Regardless of the non-neurotoxic effects produced by a MDPV dosing paradigm (similar to the METH toxic protocol used herein), our research highlights the need of further research exploring other time-points and alternative mechanistic pathways in different brain regions where MDPV may have detrimental effects.

## Figures and Tables

**Table 1:** Effect of METH and MDPV binge paradigm on mice striatal TLR protein density.

PRR ^1^	SAL	METH	MDPV
**TLR2**	100.0 ± 3.2	95.3 ± 7.0	100.6 ± 3.0
**TLR4**	100.0 ± 11.3	101.6 ± 23.1	99.1 ± 15.7
**TLR7**	100.0 ± 20.9	127.0 ± 32.8	90.7 ± 28.5

^1^ Western blot quantification of striatal toll-like receptor (TLR) TLR2, TLR4, and TLR7, group of pattern recognition receptors (PRRs). Results were normalized with GAPDH and expressed as mean % of saline (SAL) ± S.E.M. (*n* = 3–4). Data not statistically compared. MDPV, 3,4-Methylenedioxypyrovalerone; METH, methamphetamine.

## Data Availability

The data presented in this study are available in the main text.

## Abbreviations

GFAP: glial fibrillary acidic protein; TH: tyrosine hydroxylase; Iba-1: ionized calcium binding adaptor molecule; C-RAGE: receptor for advanced glycation endproducts C-terminal; N-Rage: receptor for advanced glycation endproducts N-terminal.

## Appendix A

**Table A1:** Primary and secondary antibodies used for western blot and immunohistochemistry.

Antibody	Host	Dilution	Company	Catalog Number
**Primary Antibodies**				
Anti-GFAP	mouse	1:100	Merck Millipore	IF03L
Anti-TH	rabbit	1:250	Merck Millipore	AB152
Anti-Iba-1	rabbit	1:250	Wako	019-19741
Anti-C-RAGE	rabbit	1:500	Abcam	ab3611
Anti-N-RAGE	goat	1:1000	Santa Cruz	Sc-8231
**Secondary Antibodies**				
Alexa 488, anti-mouse	donkey	1:1000	ThermoFisher^TM^	A21202
Alexa 488, anti-rabbit	goat	1:1000	Life Technologies	1124089
Alexa 594, anti-rabbit	donkey	1:1000	ThermoFisher^TM^	A21207
Alexa 488, anti-goat	donkey	1:1000	Life Technologies	A11055
